# Primary Lung Dendritic Cell Cultures to Assess Efficacy of Spectinamide-1599 Against Intracellular *Mycobacterium tuberculosis*

**DOI:** 10.3389/fmicb.2018.01895

**Published:** 2018-08-21

**Authors:** Karen Santos, Pradeep B. Lukka, Anne Grzegorzewicz, Mary Jackson, Ashit Trivedi, Fernando Pavan, Marlus Chorilli, Miriam Braunstein, Anthony Hickey, Bernd Meibohm, Mercedes Gonzalez-Juarrero

**Affiliations:** ^1^Mycobacteria Research Laboratories, Department of Microbiology, Immunology & Pathology, Colorado State University, Fort Collins, CO, United States; ^2^School of Pharmaceutical Sciences, São Paulo State University, São Paulo, Brazil; ^3^Department of Pharmaceutical Sciences, College of Pharmacy, The University of Tennessee Health Science Center, Memphis, TN, United States; ^4^Department of Microbiology and Immunology, The University of North Carolina at Chapel Hill, Chapel Hill, NC, United States; ^5^Discovery Science and Technology, RTI International, Durham, NC, United States

**Keywords:** spectinamides, *Mycobacterium-tuberculosis*, intracellular, lung, dendritic cells, GM-CSF, pyrazinamide, interferon

## Abstract

There is an urgent need to treat tuberculosis (TB) quickly, effectively and without side effects. *Mycobacterium tuberculosis* (Mtb), the causative organism of TB, can survive for long periods of time within macrophages and dendritic cells and these intracellular bacilli are difficult to eliminate with current drug regimens. It is well established that Mtb responds differentially to drug treatment depending on its extracellular and intracellular location and replicative state. In this study, we isolated and cultured lung derived dendritic cells to be used as a screening system for drug efficacy against intracellular mycobacteria. Using mono- or combination drug treatments, we studied the action of spectinamide-1599 and pyrazinamide (antibiotics targeting slow-growing bacilli) in killing bacilli located within lung derived dendritic cells. Furthermore, because IFN-γ is an essential cytokine produced in response to Mtb infection and present during TB chemotherapy, we also assessed the efficacy of these drugs in the presence and absence of IFN-γ. Our results demonstrated that monotherapy with either spectinamide-1599 or pyrazinamide can reduce the intracellular bacterial burden by more than 99.9%. Even more impressive is that when TB infected lung derived dendritic cells are treated with spectinamide-1599 and pyrazinamide in combination with IFN-γ a strong synergistic effect was observed, which reduced the intracellular burden below the limit of detection. We concluded that IFN-γ activation of lung derived dendritic cells is essential for synergy between spectinamide-1599 and pyrazinamide.

## Introduction

Tuberculosis (TB), one of the world’s oldest and deadliest pandemic diseases, is the cause of enormous suffering to humans and animals around the globe. TB adversely impacts the lives of patients, their families and communities (WHO, 2015). The health, housing-living conditions, immunity and nutritional status of infected individuals play essential roles in disease outcome. In endemic countries, TB is exacerbated by poverty and is a significant barrier to economic, social and intellectual development (WHO, 2015, 2017). There are complex and long multidrug regimen treatments available that can cure TB, however, the length, complexity and toxicity of these treatments often lead to poor patient adherence or withdrawal (WHO, 2015, 2017). The latter has contributed to the emergence and spread of multidrug resistant strains of *Mycobacterium tuberculosis* (*Mtb*) (WHO, 2015, 2017). Therefore, new, efficacious drugs allowing for shorter multidrug regimens are needed to eliminate the global menace of TB. One impediment in achieving this goal is our limited understanding of drug efficacy against intracellular bacilli.

In most instances, TB is primarily a disease of the lungs and is caused by infection with *Mtb.* The hallmark of the disease is the formation of granuloma lesions. *Mtb* resides for long periods of time within macrophages and dendritic cells and/or encapsulated within granulomas (Orme and Basaraba, 2014). Dendritic cells (DCs) are present within granuloma lesions in large numbers, and they often contain *Mtb* bacilli (Ordway et al., 2005; Ulrichs and Kaufmann, 2006; Dorhoi and Kaufmann, 2015). Dendritic cells are proposed to act as Trojan horses that provide an intracellular niche to *Mtb* for long periods of time (van Kooyk et al., 2003; Ordway et al., 2005).

The development and cell biology of macrophages and dendritic cells in the lungs is dependent on the effect of several types of Colony-stimulating factors- (CSF). CSFs are an important family of hematopoietic cytokines represented by Granulocyte-macrophage- (GM-CSF), Macrophage- (M-CSF) and Granulocyte- (G-CSF) colony-stimulating factors. GM-CSF is essential to lung myeloid cell maturation, lung microbicidal function and development of pulmonary immunity (Dranoff et al., 1994). This cytokine is known to promote cell proliferation and is commonly used to differentiate dendritic cells *in vitro* (Inaba et al., 1992). Most importantly, GM-CSF has the potential to restrict *Mtb* growth *in vitro* (Denis and Ghadirian, 1990). *In vivo*, the levels of expression of GM-CSF in the lungs during *Mtb* infection increase steadily during the acute and chronic stage of infection (Higgins et al., 2008) whereas lack of GM-CSF results in uncontrolled replication of *Mtb* in the lungs but not in spleen (Gonzalez-Juarrero et al., 2005; Szeliga et al., 2008). In steady-state conditions, epithelial type II cells produce GM-CSF but in response to *Mtb* infection natural killer T cells and conventional T cells (Rothchild et al., 2014) are important sources of GM-CSF. In our studies described below, we mimicked the lung environment during a chronic infection with *Mtb*. Myeloid lung CD11c positive cells were isolated using cell sorting and were cultured and expanded in the presence of GM-CSF.

Intracellular bacilli are difficult to eliminate with current chemotherapeutic regimens (Aljayyoussi et al., 2017). The intracellular bacilli reside within phagosomes in a low metabolic/replicative stage, also referred to as slow or non-replicative bacilli (Franzblau et al., 2012; Liu et al., 2016). Since most drugs target specific molecules and metabolic pathways in the bacilli, the efficacy of the drugs is contingent on the level of activity of the drug-targeted molecule/pathways. Thus, *Mtb* responds differentially to drug treatment depending on its extracellular or intracellular location and replicative state (Liu et al., 2016). To inhibit intracellular bacilli, drugs must be able to sequentially cross the host cytoplasmic and phagosome membranes followed by crossing of the bacterial wall and membrane (Budha et al., 2008; Schump et al., 2017). Furthermore, drugs must reach the bacilli at adequate concentrations. For the latter scenario, drug delivery to the host cell, efficacy against intracellular bacilli and intracellular drug concentration are critical parameters defining drug efficacy against intracellular mycobacteria infections (Aljayyoussi et al., 2017). However, these parameters have not been given sufficient consideration during *in vitro* *Mtb* drug screening or the early lead development process. Currently, drug screening against slow or non-replicating bacilli is performed using bacterial cultures with oxygen or nutrient deprivation, low pH, NO or a combination of low pH/NO to restrict growth (Franzblau et al., 2012). To test drug efficacy against slow or non-replicating bacilli in the intracellular compartment, tumor macrophage-derived cell lines (e.g., A549, J7774A.1, THP1 cells) and, infrequently, primary cultures of bone marrow derived macrophages or blood peripheral monocytes were used (Vogt and Nathan, 2011; Rohde et al., 2012; Liu et al., 2016; Manning et al., 2017). Although cancer derived cell lines are easy to culture and expand, these cell lines have abnormal genetics, and very rapid proliferative and/or metabolic functions. Moreover, gene expression-profiling studies show that primary macrophage cultures and cell lines (e.g., J7774A.1) differ in their responses to infection with *Mtb* (Andreu et al., 2017) and to killing by drugs (Liu et al., 2016). Thus, new approaches to isolate and culture lung macrophages and DCs are needed to study intracellular killing by anti-mycobacterial drugs. In this study using GM-CSF, we differentiated lung derived myeloid cells into dendritic like cells because both DCs and GM-CSF are abundantly present in the *Mtb* infected lung and granuloma (Higgins et al., 2008; Rothchild et al., 2014). Furthermore, DCs when differentiated in the presence of GM-CSF have only bacteriostatic activity against *Mtb* (Bodnar et al., 2001) and thus we believe this culture system is ideal to test drug efficacy against intracellular bacilli.

Spectinamide-1599 is a new semisynthetic antibiotic with demonstrated efficacy against drug susceptible and multidrug resistant strains of *Mtb* (Lee et al., 2014). In preclinical studies, spectinamide-1599 exhibits a strong antibiotic effect when administered by subcutaneous injection or intrapulmonary administration (Lee et al., 2014). Spectinamide-1599 is effective against replicating and non-replicating bacilli (Lee et al., 2014), but studies on its efficacy against intracellular bacilli have so far been limited. Pyrazinamide (PZA) is one of the first line antibiotics used for the treatment of TB with sterilizing activity (Steele and Des Prez, 1988; Ahmad et al., 2011). PZA is only active at acidic pH and has poor bactericidal activity; however, when combined with other first line drugs it accelerates sterilization (Calix and White, 1956; Girling, 1984; Steele and Des Prez, 1988). It is suggested that sterilization by PZA is related to its capacity to target subpopulations of bacilli residing in specific environments of the lungs including those within intracellular compartments (Ahmad et al., 2011). Interferon gamma (IFN-γ) is a cytokine produced by the host in response to *Mtb* infection and is essential to control bacilli load in the lungs (Flynn et al., 1993). *In vivo* studies using the murine model of TB have demonstrated synergy between spectinamide-1599 and PZA (Robertson et al., 2017).

Here, we hypothesized that combination treatment with spectinamide-1599, PZA and IFN-γ has potent bactericidal efficacy against intracellular bacilli. To test our hypothesis, we first optimized a primary lung derived cell culture system to be used as screening system for drug efficacy against intracellular bacilli. We demonstrated that cultures of lung derived DCs can sustain long-term intracellular infection with *Mtb* (H37Ra strain). This primary cell system was used to assess efficacy against intracellular *Mtb* bacilli of spectinamide-1599 and PZA when administered in the presence or absence of IFN-γ. Our results demonstrated that monotherapy with either spectinamide-1599 or PZA reduces intracellular bacilli burden, and that there is a strong synergistic effect if both drugs are administered simultaneously with IFN-γ.

## Materials and Methods

### Mice

Specific pathogen-free Balb/c female mice (Jackson Laboratories, Bar Harbor, ME, United States) of 6–8 weeks of age were used. The Colorado State University Institutional Animal Care and Use Committee approved all experimental protocols. Mice were monitored daily by trained animal laboratory technicians at Laboratory Animal Resources and researchers with experience in animal handling certified by the University.

### Bacteria

*In vitro* studies were performed with the H37Ra strain of *M. tuberculosis* (ATCC number 25177), grown from low passage seed lots in aqueous solution containing 50% Glycerol to early mid-log phase and frozen in aliquots at -80°C until needed. Aliquots were subsequently thawed and cultured with rapid stirring in 7H9 medium (Middlebrook 7H9 Broth (Millipore Sigma) supplemented with 0.05% of Tween 80 and 10% of Middlebrook ADC Enrichment] at 37°C until the optical density at 600 nm (OD_600_) reached 0.6.

### Culture Media

Dendritic cells were cultured in complete RPMI medium (cRPMI) consisting of RPMI 1640 medium (Millipore Sigma, St. Louis, MO, United States) supplemented with 1% glutamine, non-essential amino acids (Life Technologies, Grand Island, NY, United States), HEPES buffer (Millipore Sigma), sodium pyruvate (Millipore Sigma), 50 μM 2-mercaptoethanol (Millipore Sigma), 1% penicillin-streptomycin (Millipore Sigma), 10% fetal bovine serum (FBS), and 20 ng of recombinant murine granulocyte-macrophage colony-stimulating factor (GM-CSF) (PeproTech, Rocky Hill, NJ, United States) per mL. Phosphate-buffered saline (PBS) without calcium chloride and magnesium chloride (Life Technologies, Grand Island, NY, United States) was used to isolate and purify lung dendritic cells.

### Lung CD11c Positive Cell Isolation

The isolation and culture of lung CD11c positive cells was performed as previously described (Gonzalez-Juarrero and Orme, 2001). Briefly, for each isolation, two to eight Balb/c mice were euthanized and their pulmonary cavities opened. The blood circulatory system in the lungs was cleared by perfusion through the pulmonary artery with 3 mL of saline containing 50 U of heparin (Millipore Sigma) per mL. Thereafter, the lungs were aseptically removed from the thoracic cavity and placed into a petri dish containing 1 mL of cold RPMI medium. Next, the lungs were minced into small pieces using two sterile razor blades followed by incubation for 30–45 min at 37°C with RPMI medium containing collagenase XI (0.7 mg/mL; Millipore Sigma) and type IV bovine pancreatic DNase (30 μg/mL; Millipore Sigma). After this incubation period, the activity of the enzymes was stopped by diluting the minced tissue containing the enzymes with 10 mL of cRPMI followed by filtration through a 70 μm nylon screen to separate undigested tissue from the single cell suspension. The single-cell suspension was then washed and centrifuged at 200 × *g*. To lyse contaminating red blood cells, the cell pellet was incubated for 5 min at room temperature with 5 mL of Gey’s solution (NH_4_Cl and KHCO_3_). Cells were then washed with PBS containing 0.5% FBS, counted, and incubated at the appropriate ratio with MACS CD11c microbeads (Miltenyi Biotec, Auburn, CA, United States) for 15 min at 6–12°C. After being washed again with 15 mL of PBS, cells were diluted in 5 mL of PBS containing 0.5% FBS. Finally, CD11c^+^ cells were separated by passing the antibody-coated cell suspension over a VS^+^ column on a SuperMACS magnetic cell separator. Positive cells were collected by removing the column from the magnetic field and then flushing it with PBS. Isolated cells were cultured in T75 flasks at a density of ∼1 × 10^6^ cells/mL in cRPMI containing GM-CSF. Every 48 h, 5 mL of old medium was removed from the T75 flask and 5 mL of fresh cRPMI medium containing 20 ng/mL of GM-CSF was added. When cells were used for *Mtb* (H37Ra strain) infection, media was replaced with cRPMI without antibiotics. Cell cultures were imaged using an Olympus CKX41 microscope and Olympus DP camera. Images were collected and analyzed using the DP controller software.

### Cell Culture

J774A.1 cells (ATCC, Manassas, VA, United States) were cultured in T25 flasks in Dulbecco’s Modified Eagle’s Medium (DMEM; Gibco, Thermo Fisher Scientific, Waltham, MA, United States) with 10% fetal bovine serum (Sigma-Aldrich, United States) and maintained at 37°C, 5% CO_2_. Cells were scraped using Teflon cell scraper from the T25 flasks and the final cell density of 2 to 4 × 10^5^ cells/mL was adjusted using fresh DMEM. The cells were cultured for 48 h at 37°C until 60–80% confluent in a 6-well plate (each plate representing individual time point and concentration).

### Cell Density and Viability

The cell density in each culture was determined using the Cellometer Mini (Nexcelom Bioscience, Lawrence, MA, United States). On day 0, six T75 culture flasks (CELLTREAT Scientific Products, Pepperell, MA, United States) were seeded with 1 × 10^6^ cells/mL and cultured at 37°C, 5% CO_2_ during 10, 20, 30, 40, and 50 days when cells were scrapped and cell number determined as using cellometer. Cell viability in each culture and time point was monitored using Trypan blue 0.4% (Millipore Sigma).

### Flow Cytometric Analysis

Lung derived DCs from 15-day culture were washed in PBS. Monoclonal antibodies specific for CD11c (N418) or anti-Mouse CD11b were purchased from eBioscience (Thermo Fisher Scientific, Waltham, MA, United States) as direct conjugates to fluorescein isothiocyanate (FITC) or allophycocyanin (APC). Cells were stained for 60 min at 4°C with directly conjugated antibodies. Cell acquisition was performed on a FACSCanto II (BD) flow cytometer (Becton Dickinson, Mountain View, CA, United States) and data were analyzed using Flow Jo V.10.1 (FlowJo LLC, Ashland, OR, United States). The gating strategy used selected cells based on forward and size scatter followed by analysis of intensity of fluorescence for CD11c and CD11b expression. The quadrants were set based on fluorescence of unstained cell samples.

### Lung Derived DC Infection Protocol

Lung derived DCs cultured over 15 days were seeded in 12-well plates at 4 × 10^4^cells per mL of cRPMI containing GM-CSF without antibiotics. The plates were incubated for 48 h at 37°C prior to infection. In each experiment four extra wells were exclusively allocated to monitor cell numbers and viability during the study, consequently at different time points of the study, one well was used as representative sample for monitoring cell growth and viability. The bacterial inoculum was prepared by culturing H37Ra until OD_600_ = 0.6. After plating these cultures in solid agar, it was determined that an OD_600_ = 0.6 contained ∼9 × 10^6^ CFU/mL. In each study, to prepare the inoculum, an aliquot of 70 μL of H37Ra culture was centrifugated at 1300 × *g* and the pellet obtained was dissolved into 12 mL of cRPMI. Each 12-well containing ∼1 × 10^5^ cells of lung derived DCs received 1 mL of the H37Ra inoculum followed by incubation at 37°C for 4 h. Thereafter, the plates were washed twice with PBS followed by addition of fresh cRPMI containing GM-CSF without antibiotics and incubation at 37°C and 5% CO_2_. At different times post infection and post drug treatment, cells were washed, harvested and homogenized using a Bullet Blender (Next Advance, Averill Park, NY, United States). Briefly, ∼2 × 10^5^ cells/mL were placed in a 1.5 mL sterile safe lock Eppendorf tube containing 0.5 mL of sterile saline filled with sterile zirconium oxide beads (Next Advance, Averill Park, NY, United States). These tubes were homogenized in the Bullet Blender at 800 rpm during 5 min. The cell homogenate obtained was used to enumerate the bacterial load using serial dilutions and plating onto 7H11 agar plates followed by incubation at 37°C for 21 days. The bacilli burden in each sample was expressed as log_10_ of the colony forming units (CFU)/2 × 10^5^ cells.

### Acid-Fast Bacilli Staining

To determine intracellular rate of infection, lung derived DCs infected with H37Ra (4 × 10^4^ cells/mL) were citospun onto slides and stained as previously reported (Ryan et al., 2010) for acid-fast bacilli using Sybr-Gold stain [Molecular probes (Life Technologies, Eugene, OR, United States)] followed by confocal microscopy analysis. The dye was diluted 1:1000 in a stain solution consisting of phenol crystals (8 g), glycerin (60 mL), isopropanol (14 mL) and distilled water (26 mL). The staining solution was dropped generously over the slide. The slides were heated on a block at 65°C for 5 min and then allowed to cool for 1 min at room temperature. Thereafter, the slides were washed in acid alcohol (0.5% HCL, 70% isopropanol) for 3 min, then washed with water and mounted using Prolong Gold antifade mounting medium with DAPI. Samples were analyzed using a Zeiss LSM 510 confocal microscope equipped with the Zen 2009 software (Zeiss).

### Drug Treatment

Spectinamide-1599 was synthesized as described (Lee et al., 2014) and provided by Dr. Richard Lee (St. Jude Children’s Research Hospital). Pyrazinamide, spectinomycin and streptomycin were purchased from Sigma-Aldrich (Millipore Sigma, St. Louis, MO, United States) and recombinant murine Interferon Gamma (IFN-γ) from PeproTech (Rocky Hill, NJ, United States). Stock solutions for each drug were prepared as follow; Spectinamide-1599 was diluted at 300 μg/mL with cRPMI Medium. Pyrazinamide was dissolved in cRPMI medium at 20 mg/mL and IFN-γ was dissolved in cRPMI medium at 300 U/mL. On day 2 post-infection cell cultures received drug treatment. Cell cultures were treated with monotherapy or combinations of 200 or 400 μg/mL of PZA, 25 or 100 μg/mL spectinamide-1599 and 50 U/mL murine IFN-γ.

### Cell Cytotoxicity

Cytotoxicity was assessed using the Resazurin microtiter assay. Resazurin (Cell Signaling Technology, Danvers, MA, United States) is a non-fluorescent blue dye reduced by metabolically active cells to the pink and fluorescent resorufin dye. Initially, 100 μL dendritic cell suspension containing 2.5 × 10^5^ cells/mL was plated in 96-well plates and incubated for 24 h at 37°C until adherence of the cells. Thereafter, the culture medium was removed and the cells were treated for 24 h with 200 μL/well of cell culture media containing 10 or 20 μg/mL of spectinamide-1599; 200 or 400 μg/mL of PZA and 50 U/mL of IFN-γ. After 24 h of treatment, each culture received 10 μL of resazurin solution, followed by incubation at 37°C for 3 h. The plates were read on a fluorescence reader (Synergy H1, BioteK^®^, Winooski, VT, United States) using wavelengths of 530 nm (excitation) and 590 nm (emission).

### Drug Uptake

The lung derived DCs were plated (12-well plates) at 4 × 10^4^ cell/mL and incubated for 48 h when cells were infected as above. Forty-eight hours after infection, the spectinamide-1599 (25/100 μg/mL) was added to each treatment well and cultured for an additional 72 h. Subsequently, these cells were washed twice with PBS. Duplicate samples obtained by combining cells from six wells each were homogenized as above using PBS. A total of 1 ml of PBS was collected from each replicate and frozen immediately at -80°C. The cells were suspended in 1 mL of water and sonicated for 2 min. Cultures of J774A.1 were cultured with 25 or 100 μg/mL spectinamide-1599, spectinomycin or streptomycin for 24 h. Similar cultures of J774A.1 cells were treated with spectinamide-1599 (25 or 100 μg/mL) and samples were collected after 0.5, 1, 2, 4, 8, and 24 h. Cell suspensions were centrifuged for 5 min at 10,000 × *g* and the supernatant collected was used to determine the drug uptake using an LC-MS/MS assay. The drug uptake was expressed as pg/per cell.

### Drug Quantification by LC-MS/MS

All specimens for drug quantification underwent protein precipitation by addition of 100 μL of internal standard solution (spectinamide-1329; 10 ng/mL in methanol) to 25 μL of sample. After vortexing and centrifugation at 10,000 × *g* and 4°C for 10 min, the supernatant was chromatographically separated using a Shimadzu Nexera XR (LC-20ADXR) liquid chromatograph (Shimadzu Corporation, United States) consisting of two pumps, online degasser, system controller and an auto sampler. A mobile phase consisting of (a) water with 5 mM ammonium formate buffer and (b) methanol with 5 mM ammonium formate buffer was used at a flow rate of 0.4 mL/min in gradient mode. A Phenomenex HILIC 3.5 mm, 100 mm × 4.6 mm column (Phenomenex, United States) was applied for the separation. Samples (10 μL) were injected on column and the eluate was led into an API 4500 triple quadruple mass spectrometer (Applied Biosystems, Foster City, CA, United States) equipped with a turbospray ion source operated in positive ion mode. The characteristic mass transfers for 1599 (487.2 → 207.1) and internal standard (453.0 → 247.1) were monitored in multiple reaction monitoring mode with a declustering potential of 45 V and collision energy of 25 eV. Data were acquired and processed with Analyst software version 1.6.2 (Applied Biosystems, Foster City, CA, United States). Spectinamide-1599 concentrations were determined by comparing peak area ratios between 1599 and internal standard for unknown samples to a previously established calibration curve. The accuracy was within ±5.1% over the entire range of the calibration curve, and the precision (coefficient of variation) of the assay was <1.5%, with a lower limit of quantitation of 0.976 ng/mL.

### Statistics

One-way analysis of variance was used for analyzing the data by comparing all the groups to each other at the confidence interval of 95%. Calculations were performed using GraphPad Prism version 7.00 (San Diego, CA, United States). *P*-values < 0.05 were considered significant.

## Results

**FIGURE 1:**
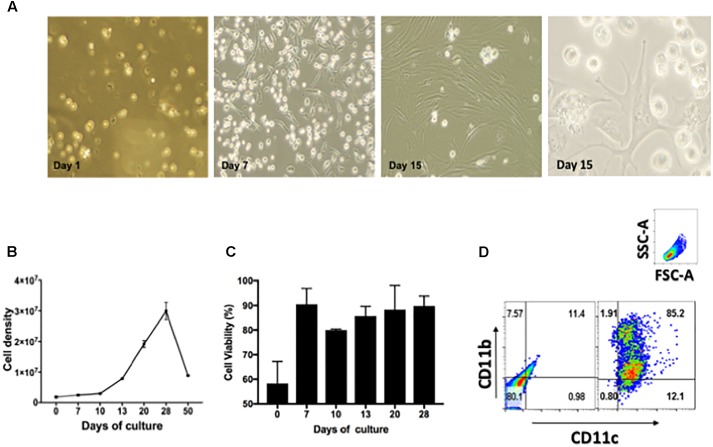
Characterization of lung derived dendritic cell cultures. **(A)** Phase contrast microscopy of CD11c positive cells isolated from lungs of Balb/c mice and cultured during 1, 7, and 15 days in the presence of 20 ng/mL GM-CSF. Media was replenished every 48 h. **(B)** Cell density expressed as total cell number/T75 flask after culturing isolated CD11c positive cells during 0, 10, 20, 30, 40, and 50 days in the presence of GM-CSF, *p* > 0.05. **(C)** Cell viability of CD11c cultures expressed as percentage of viable cells after culturing 0, 7, 10, 13, 20, and 28 days in the presence of GM-CSF, *p* > 0.05. **(D)** Phenotype of CD11c positive cells after cultured as above for 15 days were stained with fluorescence labeled mAb CD11b/CD11c and analyzed by flow cytometry. The gating strategy used selected cells based on forward and size scatter area (FSC-A/SSC-A) followed by analysis of intensity of fluorescence for CD11c and CD11b expression. The quadrants were set using unstained cells (left dot-plot). Cells stained for CD11c and CD11b are shown in the right dot-plot. Numbers in each quadrant represent the percentage of positive cells. Representative data from more than three experiments in similar conditions.

To demonstrate drug efficacy against intracellular bacilli, this study optimized the isolation and culture conditions of lung derived CD11c positive cells and their intracellular infection with *Mtb* using a previously reported method (Gonzalez-Juarrero and Orme, 2001). Thus, cell suspensions of lung CD11c positive cells were cultured in the presence of cRPMI containing 20 ng/mL of recombinant murine GM-CSF during 1, 7, 15, and 30 days. **Figure 1A** shows representative images obtained by phase contrast microcopy of changes in the morphological features of cells over 30 days of culture. Cells transitioned from a single spherical morphology to adherent and non-adherent cells with long cytoplasmic projections (**Figure 1A**). The number and viability of cells significantly increased over time during the first 21 days of culture, and decreased thereafter [**Figure 1B** (*p* > 0.05) and **Figure 1C** (*p* > 0.05), respectively]. Flow cytometry studies demonstrated that the cell surface phenotype during 15 days of culture is that of CD11c+/CD11b+ (**Figure 1D**). As reported previously, when lung CD11c isolated cells are cultured in the presence of GM-CSF, they proliferate and differentiate into cells resembling a dendritic cell type.

**FIGURE 2:**
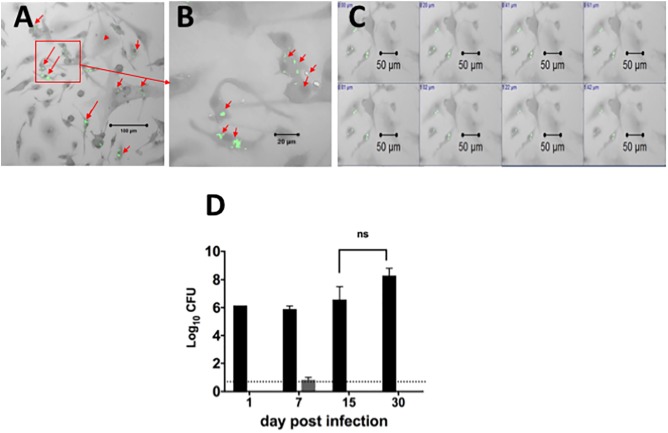
Localization and quantification of intracellular *Mtb* bacilli in lung derived DCs. Lung derived DC cultures were infected with *Mycobacterium tuberculosis* (H37Ra strain) as explained in Section “Materials and Methods.” Cells were washed and cultured with cRPMI media (without antibiotics) containing GM-CSF as in **Figure 1**. Acid fast positive bacilli within cells were visualized using a confocal ZEISS LSM 510 microscope after staining of lung DC cells by the acid fast staining method with Sybr Gold (green). **(A)** Intracellular H37Ra bacilli in lung dendritic cell cultures after 7 days of infection. **(B)** Magnification of picture in **(A)**, showing cells containing one, two or many bacilli. **(C)** Demonstration of intracellular location of bacilli in a confocal imaging *Z*-stack of cells scanned at 0.2 μm steps. **(D)** The *Mtb* bacilli burden in lung dendritic cell homogenates infected for 1, 7, 15, and 30 days (black bars) in similar cultures of lung DCs infected with *Mtb* as in **(A)**. The *Mtb* bacilli burden in supernatants from each cell culture is represented in gray bars. The number of colony forming units (CFU) is expressed as log_10_ CFU. The results suggest that almost all bacteria are located intracellularly, and that there is no significant change in the number of intracellular bacteria over the observation period. The dotted line represents the detection limit of the assay. Representative data from more than three experiments in similar conditions.

Since our goal was to develop a system for assessing efficacy of drugs against intracellular bacilli in primary DCs, we also optimized infection of lung derived DC cultures with *Mtb* (H37Ra strain). Forty-eight hours prior to infection, cells were cultured in media containing GM-CSF without antibiotics, and were then exposed to the bacterial inoculum. The non-adhered bacilli were removed from culture and cells were cultured again in the presence of culture media containing GM-CSF but without antibiotics. The cells were examined for the presence of acid fast positive bacilli using confocal microscopy at 1, 7, 15, and 30 days after infection and culture, to assess intracellular location of bacilli (**Figure 2**). As shown in **Figures 2A,B**, the acid fast positive bacilli were associated with cells and there were no extracellular bacilli. Individual cells contained different numbers of bacilli ranging from one to several bacilli (**Figure 2B**). Furthermore, the intracellular location of the bacilli was confirmed by microscopically scanning cells every 0.2 μm (**Figure 2C**). This analysis demonstrated that the acid fast positive bacilli were present in each plane of the stack indicating intracellular localization of the bacilli.

The viability of intracellular *Mtb* in DCs was determined by quantitating the bacterial burden in DCs over time. Cell homogenates or supernatants from lung derived DC cultures infected for 1, 7, 15, and 30 days were plated on agar to determine the numbers of viable colony forming units (CFU) over time (*p* > 0.05, **Figure 2D**). The results indicated that bacilli recovered from these lung derived DC cultures formed colonies on the agar, indicating viable intracellular bacilli. The number of bacilli recovered was the same for the first three time points (1, 7, and 15 days of infection) and increased thereafter; however, the differences were not statistically significant. Furthermore, the number of CFU in supernatant from cultures of lung DC, except for Day 7, was below the detection limit for the assay. We conclude that infection of primary cultures of lung derived DCs with *Mtb* results in intracellular bacilli that persist over extended periods of culture. In addition, this study demonstrates that lung derived DCs sustain long-term infection with *Mtb* and suggests that bacilli within these cells remain in a slow or non-replicative state or that there is equilibrium between bacilli growth and kill rate.

**FIGURE 3:**
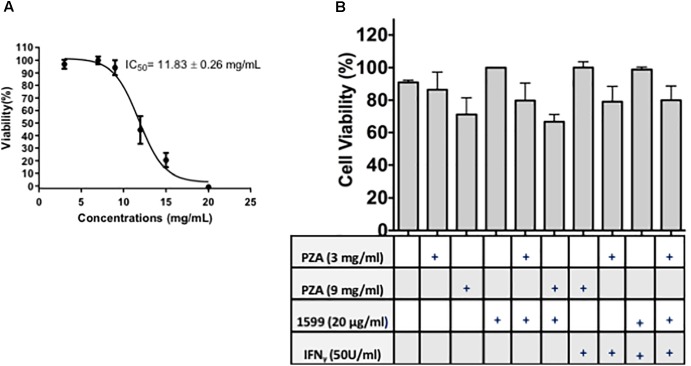
Cell viability of lung derived DCs after drug treatment. **(A)** The resazurin colorimetric assay was used to determine the half maximal inhibitory concentration (IC_50_) for pyrazinamide on lung dendritic cell cultures. **(B)** Cell viability of lung derived DC cultures after 24 h of incubation with cRPMI media containing GM-CSF and single drug treatment of PZA at 3 mg/mL or 9 mg/mL, spectinamide-1599 (20 μg/mL) or IFN-γ (50 U/mL). Statistical analysis revealed no significant difference among the groups (*p* > 0.05).

Spectinamide-1599 and PZA demonstrated potent synergy in *in vivo* studies using murine models of TB infection (Robertson et al., 2017). In this investigation, we expanded on these findings and assessed the efficacy of spectinamide-1599 against intracellular bacilli and its synergy *in vitro* with PZA. Furthermore, because IFN-γ is a cytokine essential to myeloid cell function and present in the host during chemotherapy administration, we also assessed the efficacy of these drugs in the presence and absence of IFN-γ. Firstly, we determined if there is a toxic effect of the drug on the lung derived DCs and thus, the cells were cultured during 24 h in the presence of serial dilutions of 20, 15, 10, 5, or 0 mg/mL of PZA followed by assessment of cell viability using the resazurin staining method. Exposure of PZA up to 11.83 mg/mL resulted in 50% or better cell viability. At higher concentrations <50% cell viability was observed (**Figure 3A**). In a similar study, treatment of lung derived DCs with PZA at 3 and 9 mg/mL demonstrated 80 and 90% cell viability while treatment of similar cultures with spectinamide-1599 (20 μg/mL) or IFN-γ (50 U/mL) or higher did not affect cell viability (*p* > 0.05; **Figure 3B**). When lung derived DCs were cultured with combinations of spectinamide-1599 (20 μg/mL) and PZA (3 and 9 mg/mL), PZA caused a concentration-dependent decrease in cell viability that was independent of the presence or absence of spectinamide-1599. Moreover, addition of IFN-γ (50 U/mL) to similar drug combinations did not affect cell viability.

**FIGURE 4:**
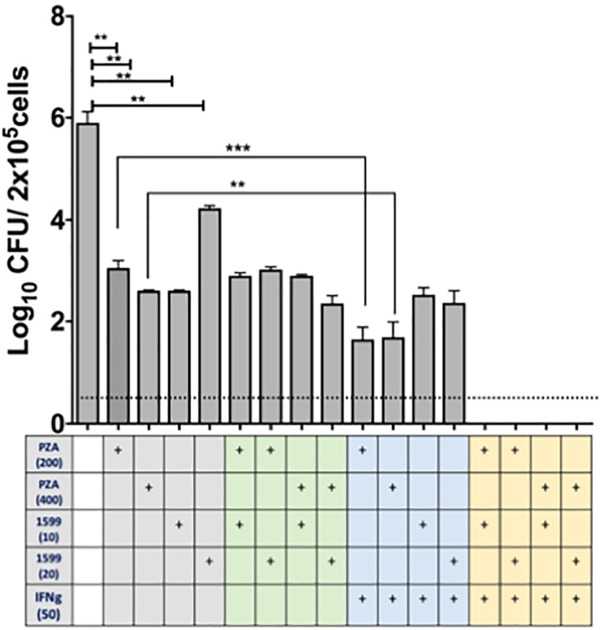
*In vitro* efficacy of spectinamide-1599, PZA, and IFN-γ against intracellular *Mtb* hosted by lung derived DCs. Lung derived DC cultures were infected with *Mtb* (H37Ra strain) as explained in the Section “Materials and Methods.” Cells were treated with mono, double or triple combinations (*X*-axis) of PZA (200 or 400 μg/mL), spectinamide-1599 (10 or 20 μg/mL), murine recombinant IFN-γ (50 U/mL) for 7 days. The cell culture media and drugs were replaced every 48 h. Cell homogenates were plated on agar 7H11 and cultured for 3 weeks at 37°C. The number of colony forming units (CFU) is expressed as log_10_ CFU/5 × 10^5^ cells (*Y*-axis). Statistical analysis revealed significant difference (*p* < 0.05) when untreated samples were compared to mono-therapy treatments and for combinations of PZA versus PZA plus IFN-γ. Representative data from a large experiment and two pilot experiments in similar conditions. ^∗∗^*p* < 0.01; ^∗∗∗^*p* < 0.001.

The efficacy of spectinamide-1599 and PZA against intracellular bacilli in lung derived DCs was also studied. Cultures of lung derived DCs infected with *Mtb* were treated in triplicate during 7 days with spectinamide-1599 (10 or 20 μg/mL), PZA (200 or 400 μg/mL), alone or in combination (**Figure 4**). The cRPMI media containing GM-CSF and drugs were replaced every 48 h and at the end of the culture, media was removed and the cells were homogenized as explained in the Section “Materials and Methods.” Serial dilutions of each homogenate were plated on 7H11 agar plates and CFU counted. The results showed that cells treated with spectinamide-1599 or PZA alone or in combinations reduced the bacterial burden by more than 3.5 log_10_ CFU (*p* < 0.05).

The host impact on drug efficacy against intracellular bacilli was measured via addition of IFN-γ (50 U/mL) to lung derived DC cultures infected with *Mtb*. As shown in **Figure 4**, when PZA was administered simultaneously with IFN-γ there was enhanced reduction in CFUs (*p* < 0.05). However, this effect was not observed when infected cells were treated simultaneously with spectinamide-1599 and IFN-γ. Most interestingly, the combination of PZA, spectinamide-1599 and IFN-γ reduced the bacterial burden in lung derived DCs infected with *Mtb* to below the detection limit of the assay (0.4 CFU/mL). We conclude that both PZA and spectinamide-1599 can reduce more than 99.9% of intracellular bacilli on their own but simultaneous addition of IFN-γ to the drug cocktail reduces bacterial burden below the detection limit of the assay.

**FIGURE 5:**
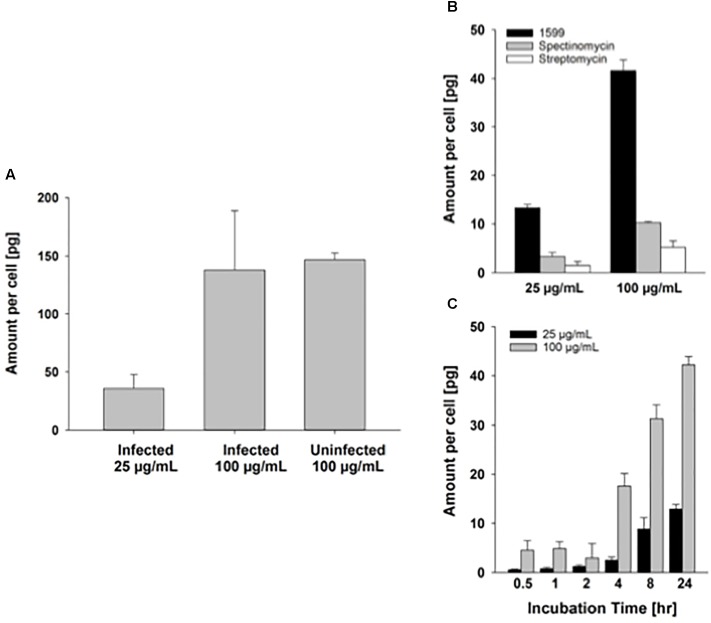
Intracellular uptake of spectinamide-1599 in lung derived DCs and J774A.1 cells. **(A)** Amount of spectinamide-1599 per cell in lung derived DCs that were either uninfected or infected with *Mtb* after incubation with 25 or 100 μg/mL spectinamide-1599 for 72 h. **(B)** Amount of spectinamide-1599 per cell after incubation of J774A.1 cells with 25 or 100 μg/mL spectinamide-1599, spectinomycin or streptomycin for 24 h. **(C)** Amount of spectinamide-1599 per cell after incubation of J774A.1 cells with 25 or 100 μg/mL spectinamide-1599 for 0.5, 1, 2, 4, 8, and 24 h.

Intracellular delivery of drugs to cells is a prerequisite for antibiotic activity against intracellular bacilli. As PZA has been shown to penetrate well into murine macrophages (Acocella et al., 1985), we focus here on spectinamide-1599. A previous study suggested that spectinamides have good permeability across murine cell membranes (Madhura et al., 2016). Our preliminary results on the uptake of spectinamide-1599 in infected and uninfected murine lung derived DCs after treatment for 72 h with 25 or 100 μg/mL are in line with this observation (**Figure 5**). First, there does not seem to be a difference in the amount taken up by infected compared to uninfected lung derived DCs (**Figure 5A**). Second, the difference in the uptake between 25 and 100 μg/mL is 3.9-fold, as expected based on the concentration difference. This observation is similar to the magnitude of difference for drug uptake into J774A.1 cells (**Figures 5B,C**), a Balb/c derived monocyte macrophage cell line where the uptake difference was 3.3-fold for the same concentrations in our previous studies. These studies suggest that the state of infection of the lung derived DCs does not affect uptake of spectinamide-1599 and that there is a direct relationship between drug exposure and intracellular concentrations.

## Discussion

This study details the optimization of lung myeloid CD11c positive cells to assess efficacy of drugs against intracellular *Mtb* bacilli. We reasoned that lung derived myeloid cells differ from similar cells in other tissues (Scott et al., 2014). In the lungs, GM-CSF regulates differentiation, activation and expansion of alveolar macrophages, alveolar type II epithelial cells and dendritic cells; however, this cytokine is not essential for similar cell functions in other tissues (Dranoff et al., 1994). *Mtb* mainly establishes infection in the lungs and current chemotherapy against TB targets free living and intracellular bacilli, but drug efficacy against intracellular bacilli is not well understood (Franzblau et al., 2012; Liu et al., 2016). Thus, it is essential to improve our models for testing drug efficacy against intracellular *Mtb* and we believe the use of a lung-derived cell model as described here is an important contribution toward this goal.

We show that lung myeloid CD11c positive cells when cultured in the presence of recombinant murine GM-CSF undergo cell proliferation and differentiation toward a DC phenotype. These cultures sustain long-term (over 15 days) intracellular infection with *Mtb* (H37Ra strain) as the bacilli burden in these cultures remained unchanged for 30-days post infection. Our results using lung derived DCs are consistent with other studies suggesting that murine bone marrow DCs and human blood DCs differentiated in the presence of GM-CSF develop only bacteriostatic activity against *Mtb* (Bodnar et al., 2001; Vogt and Nathan, 2011). In contrast, other studies using cultures of bone marrow or blood derived macrophages infected with *Mtb* had a steady increase in intracellular bacilli number during extended culture (Vogt and Nathan, 2011; Liu et al., 2016). In this study, however, lung derived DC were infected with the avirulent strain H37Ra, and future studies need to determine if the ability of the cells to control growth of the intracellular *Mtb* differs when virulent strains or clinical isolates are used to infect the lung derived DCs. Thus, differences in the source of cell progenitors (lung versus bone marrow or blood), bacterial strains (attenuated or virulent) and culture conditions (GM-CSF versus L-929 conditioned media or M-CSF) may account for the differences observed in growth of intracellular bacilli.

The plateau in intracellular bacilli burden in our lung cell system suggests that either bacilli within lung DCs remain in a non-replicative state or that there is equilibrium between growth and kill rate of the intracellular bacilli. This is important information because, acid fast staining and microscopy revealed cells with one, two or more bacilli. It will be important to determine whether all of these intracellular bacilli are alive. Others have suggested that intracellular *Mtb* in macrophage cultures are killed more slowly by first line drugs (Aljayyoussi et al., 2017). A few high throughput microscopy methods capable of visualizing dead and live bacteria were developed to measure the killing kinetics of intracellular bacilli (Sorrentino et al., 2016; Aljayyoussi et al., 2017; Manning et al., 2017). In the future, we plan to apply these methodologies to drug killing of intracellular bacilli located within lung derived DCs.

Lung derived DCs infected with *Mtb* (H37Ra) were used to assess intracellular bacilli susceptibility to two drugs with reported efficacy against non-replicative *Mtb* bacilli (Ahmad et al., 2011; Lee et al., 2014). Here it is shown that both spectinamide-1599 and pyrazinamide (PZA) can reduce the intracellular bacilli load by more than 99.9% when *Mtb* infected lung derived DCs are treated with 10 and 20 μg/mL of spectinamide-1599 or 200 and 400 μg/mL of PZA. The bacterial killing of either drug alone was similar and no differences were observed between drugs or drug concentrations tested. Furthermore, both drugs (in the absence of cell stimulation by IFN-γ) had no synergistic efficacy, as there was no enhanced killing when *Mtb* infected lung derived DCs were treated simultaneously with both drugs.

The primary lung DC system was also used to assess efficacy against intracellular *Mtb* bacilli of spectinamide-1599 and PZA when administered simultaneously with IFN-γ. Simultaneous administration of spectinamide-1599 with IFN-γ did not affect killing of bacilli in the culture beyond that observed for treatment with spectinamide-1599 alone. Nonetheless, when lung derived DCs infected with *Mtb* were treated simultaneously with PZA and IFN-γ, there was enhanced killing activity beyond that provided by PZA alone, although this effect was not sufficient to eliminate all bacilli in the cell culture system. PZA has been described as the “miracle” drug capable of accelerating sterilization in TB patients. It was added to the standard multidrug TB regimen along with isoniazid, rifampicin, and ethambutol because its inclusion in this regimen reduces the necessary length of TB patient chemotherapy treatment from 9 to 6 months (Steele and Des Prez, 1988; Ahmad et al., 2011; Lanoix et al., 2016). It was suggested that PZA targets specific subpopulations of bacilli including those within the intracellular space but it is only active at acidic pH. We speculate that *Mtb* within resting lung derived DC cultures reside in non-acidic phagosomes but when treated with IFN-γ these phagosomes acidify (Vandal et al., 2008; Herbst et al., 2011). Therefore simultaneous addition of PZA and IFN-γ would facilitate PZA efficacy against intracellular *Mtb* by altering the environmental pH encountered by the bacilli. However, the exact mechanisms governing these events remain to be studied.

A striking observation was made when spectinamide-1599, PZA and IFN-γ were simultaneously added to the lung derived DCs cultures. This drug combination resulted in no observable CFUs on agar plates and we inferred that IFN-γ activation of the lung derived DCs is essential for synergy between spectinamide-1599 and PZA. These results concur with a previous *in vivo* study in mice showing synergy of spectinamide-1599 and PZA (Robertson et al., 2017). *In vivo*, IFN-γ is present in the lungs during *Mtb* infection and may explain the observed synergy between PZA and spectinamide-1599. Further studies will determine the exact mechanism(s) of action for the observed synergy between these two drugs when combined with IFN-γ.

Here we show enhanced killing of *Mtb* when IFN-γ is present in a culture containing spectinamide-1599 and PZA. On the contrary, others have observed (Liu et al., 2016) that activation with IFN-γ and LPS of the macrophages in *in vitro* tissue culture infections induces drug tolerance in *Mtb* (Liu et al., 2016). IFN-γ is often considered the principal macrophage-activating factor (Nathan et al., 1983) and its synergistic effect with anti-TB drugs may be dependent on the time of its addition, its concentration, and the drug combination tested. It is also possible that differentiation of myeloid cells in the presence of GM-CSF or M-CSF may affect drug efficacy upon IFN-γ activation. Thus, overall differences in experimental design between studies may account for contrasting results.

Drug efficacy against intracellular bacilli is dependent on the drug’s ability to sequentially cross the cytoplasmic, phagosome and bacterial membranes. As a prerequisite for intracellular activity, adequate concentrations of the drug in the intracellular compartment are necessary to eliminate the bacilli. As previously reported PZA penetrates well into murine macrophages (Acocella et al., 1985), and thus we focused our preliminary experiments on determining the exact intracellular quantities of spectinamide-1599 in lung derived DCs and whether they are affected by the presence or absence of intracellular bacilli. It was noted, that there was no difference in intracellular uptake for spectinamide-1599 between cells carrying or not carrying the intracellular bacilli. This is an important observation as cell infection with *Mtb* has been suggested to at least affect the uptake of some classes of therapeutic agents (Rosenblatt et al., 2000). Furthermore, the amount of drug accumulated inside the lung derived DCs was proportional to the concentration of drug added to the culture. Future studies will expand on these findings to determine co-concentrations of spectinamide-1599 and PZA when added together. An important observation will also be to test the effect of IFN-γ on intracellular concentrations of these drugs. As mentioned above we also noticed that some cells support intracellular infection of more than one bacilli, raising the question of whether drug concentrations in each cell will need to be adjusted depending on the number of intracellular bacilli.

## Conclusion

Lung derived dendritic cells were used as a screening system for drug efficacy against intracellular mycobacteria. Cultures of lung derived dendritic cells differentiated and proliferated in the presence of GM-CSF and these cells supported long-term intracellular infection of Mtb. Spectinamide-1599 and pyrazinamide (antibiotics targeting slow-growing bacilli) were able to reduce the intracellular burden. Interestingly, spectinamide-1599 and pyrazinamide had strong synergy against intracellular mycobacteria when administered simultaneously with IFN-γ, but not when used in the absence of IFN-γ. Future work focused on intracellular drug concentrations and mechanism of action for IFN-γ synergy are warranted.

## Author Contributions

KS and MG-J conceived and designed the project, analyzed and wrote the manuscript. KS, MJ, and AG provided assistance with bacterial growth and infections. PL, AT, and BM performed PK studies, analyzed and interpreted data for PK. FP, MC, MB, AH, and MG-J revised the manuscript and provided funding. All authors have read and approved the final version of the manuscript.

## Conflict of Interest Statement

The authors declare that the research was conducted in the absence of any commercial or financial relationships that could be construed as a potential conflict of interest.
